# Immunodeficiency Accelerates Vitamin A Deficiency

**DOI:** 10.1093/cdn/nzab129

**Published:** 2021-10-27

**Authors:** Luigi M De Luca, Victoria Hill Petrides, Nadine Darwiche, Laura Armey, Amanda Palmer, Keith P West

**Affiliations:** Laboratory of Cellular Carcinogenesis and Tumor Promotion, National Cancer Institute, Bethesda, MD, USA; Center for Human Nutrition, Department of International Health, Johns Hopkins Bloomberg School of Public Health, Baltimore, MD, USA; Independent Consultant, Bethesda, MD, USA; Laboratory of Cellular Carcinogenesis and Tumor Promotion, National Cancer Institute, Bethesda, MD, USA; Biochemistry and Molecular Genetics, American University of Beirut, Beirut, Lebanon; Naval Postgraduate School, Monterey, CA, USA; Center for Human Nutrition, Department of International Health, Johns Hopkins Bloomberg School of Public Health, Baltimore, MD, USA; Center for Human Nutrition, Department of International Health, Johns Hopkins Bloomberg School of Public Health, Baltimore, MD, USA

**Keywords:** vitamin A deficiency, athymic mice, SENCAR (SENsitive to CARcinogenesis) mice, immunodeficiency, retinol, retinyl palmitate

## Abstract

**Background:**

Vitamin A deficiency increases susceptibility to infection caused by impaired immune function.

**Objectives:**

We investigated whether immunodeficiency could facilitate the development of vitamin A deficiency.

**Methods:**

Vitamin A deficiency was followed in 2 mouse models of immunodeficiency: the athymic nude mouse (*nu/nu*) and the humoral immunodeficient SENCAR (SENsitive to CARcinogenesis) mouse. Vitamin A deficiency was also monitored in outbred Balb/c and in NIH mice. The monitoring of vitamin A deficiency was done after feeding the mice and their mothers a semisynthetic, vitamin A–deficient diet from birth of the experimental mice. These mice were weaned onto the same deficient diet at 3–4 wk of age, while control groups were fed the same diet containing 3 μg retinoic acid per gram of diet.

**Results:**

The immunodeficient *nu/nu* and SENCAR mice developed vitamin A deficiency earlier than either the heterozygous *nu/+* controls or the Balb/c and NIH strains. In female mice, symptoms included depletion of liver retinol and retinyl palmitate, squamous metaplasia of the uterus, and death. Male mice lost weight more frequently and sooner than female mice, in which mortality generally occurred in the absence of loss of body weight. Pairwise comparisons using Tukey's honest significant difference test of the *nu/nu* and SENCAR mice versus the Balb/c and NIH mice showed a faster loss of retinol and retinyl palmitate in all pairs (*P *≤ 0.0001) except for retinol when comparing *nu/nu* and NIH strains (*P *= 0.3383).

**Conclusions:**

Our findings are consistent with an increased usage of liver retinol and retinyl palmitate in the immunocompromised *nu/nu* and in the immunodeficient SENCAR mice and suggest that compensatory mechanisms dependent on vitamin A utilization are called upon to rescue immunodeficiency both in the T-cell–deficient phenotype of the *nu/nu* mice and in the humoral immunodeficient SENCAR mice.

## Introduction

Immunodeficiency is a public health problem deriving from genetic, environmental, and nutritional causes. It is known that NK cell deficiency is important in patients with congenital immunodeficiency. Several genes have been identified as etiological agents among some 40 genetically defined congenital immunodeficiency diseases found to impair the function of NK cells (1, 2). Patients with asthma exhibit increased risks of other infections (3), implicating some interference of impaired host defense mechanisms as underlying causes. In adults with chronic obstructive pulmonary disease, there may be an underlying primary immune deficiency. Immunoglobulin G (IgG) replacement therapy has been suggested as a treatment after diagnosing antibody deficiency disease (4).

In asthma, the presence of T cells polarized to a T-helper 2 (TH2) phenotype and/or macrophages polarized to an M2 phenotype that have reduced capacity for cell-mediated immunity is thought to be a cause of increased susceptibility (4).

The environment has been ascribed roles in mediating immune responses. Skin microbiota strongly influence immunity, orchestrating the maturation of immune cells (5). Poisonous chemicals, whether airborne or systemically ingested, also affect the function of immune cells. Arsenic interferes with the functions of dendritic cells derived from human monocytes (6). Infection is of primary importance as an environment-born exposure conditioning immune response.

Diet and nutrition are implicated in the development, maintenance, and responsiveness of the immune system. A summary of the effects of micronutrients on the immune response has been published (7).

Vitamin A deficiency in humans (8) and in animals (9, 10) has consequences for immune defenses and increased susceptibility to viral and bacterial infections. Rats fed a vitamin A–deficient diet under germ-free conditions can live for as long as 272 d, whereas littermates fed the same vitamin A–deficient diet in a conventional animal room survived no longer than 54 d (11), suggesting vitamin A usage is diminished under germ-free conditions. Vitamin A–deficient animals also show depressed humoral (12, 13) and cellular immunity (14, 15) and decreased resistance to experimental infections (16).

We took advantage of the naturally athymic status of the nude mouse to study the effect of immunodeficiency on the onset of vitamin A deficiency. It has been reported that mutations in the nude*locus* produce hairlessness and athymia (17, 18). The *nu/nu* mouse contains normal hair follicles at birth but the hair shaft coils and fails to penetrate the epidermis (19). The genetic causation of this phenomenon apparently is a spontaneous loss-of-function mutation resulting in a recessive homozygosity in the locus of forkhead box (*Fox*) *N1*, encoding several transcription factors (20). It is known that the nude mutation segregates as a single autosomal locus on mouse chromosome 11 (21, 22). The mice with athymia lack T lymphocytes and are used in cancer research because they easily accept tumor tissue from other species, thereby permitting the study of different drugs as possible inhibitors of human cancer cell growth (19). The nude *locus* resides in a region enriched approximately 3-fold for nucleotide sequences of which 20–25% are transcribed. There is no evidence for any retinoid metabolizing/regulatory gene in that locus. Vascularization of the thymus and colonization by T-cell progenitors, as well as their selection, all depend on *Fox N1* (23).

We also used the SENCAR (SENsitive to CARcinogenesis) mouse, a model used widely to study the effectiveness of different biological and synthetic compounds as initiating or tumor-promoting agents (24). Its susceptibility to chemical carcinogens and tumor promoters (25) may be related to its immunodeficiency, which specifically affects class switching to T-independent antigens (26).

## Methods

### Materials

All-*trans* retinol, retinyl palmitate (RP), and BHT were from Sigma Chemical Co.; and (15–^3H^)-retinol (specific radioactivity, 29.5 Ci/mmol, 105.9 × 10^10^ Bq/mmol) was from Dupont NEN Research Products.

HPLC-grade acetonitrile, methylene chloride, and methanol were from Burdick and Jackson, HPLC-grade hexane from Pierce Chemicals, 1-octanol (certified) from Fisher Scientific Co., and diethyl ether was from Fluka. All solvents used for HPLC were filtered through a 0.2-μm nylon filter, and all procedures were carried out under yellow light to protect retinol and its derivatives from isomerization and oxidation.

### Animals and diets

Pregnant female mice of all strains were obtained from the National Cancer Institute–Federally Funded Research and Development Center Animal Production (NCI-FFRDC; Frederick, MD). Athymic NCr-nu mice were originally derived from founder mice obtained by the NCI from Dr. CW Friis's colony in Denmark. Taconic (Gaithersburg, NY) received the NCr nude spontaneous mutant model from NCI in 1993 after several years of random breeding. The mice were derived by hysterectomy to achieve germ-free status. Although initially deemed to be a Balb/c congenic, it was then determined to have a Balb/c inbred and NIH(S) outbred stock in its genetic background.

Procedures described in the Public Health Service Policy on Humane Care and Use of Laboratory Animals (Policy) and the Guide for the Care and Use of Laboratory Animals (Guide) were followed. The NIH Animal Care and Use Committee approved our protocols. For successful breeding, heterozygous females (*nu/+*) were mated to homozygous males (*nu/nu*). Litters usually contain 50% *nu/nu* and 50% *nu/+* phenotypes. Litters were culled to 8 females or males per dam after randomization for each sex and cross-fostering. Phenotypes were easily recognized by the presence or absence of the fur coat. Mice were housed 4 per polycarbonate mouse cage on heat-treated hardwood chips.

Pregnant female SENCAR mice were also procured from NCI-FFRDC Animal Production, Frederick, MD. Litters were culled to 8 females or males per dam. Mice were housed 4/5 per polycarbonate mouse cage on heat-treated hardwood chips. Water and diets were available ad libitum. Each mouse was uniquely identified by the assignment of a consecutive animal number and identified by ear mark.

To induce vitamin A deficiency, pregnant mice from all strains were fed the vitamin A–deficient diet (TD85239; Teklad) and their offspring were weaned onto the same diet at 3–4 wk of age. This protocol permits to obtain vitamin A deficiency within 15 to 20 wk from birth (27). Control mice and their mothers were fed the same diet supplemented with 3 μg retinoic acid (RA) per gram. If the mice were kept on a normal RP-containing diet until weaning of the experimental mice, the onset of vitamin A deficiency is not observed until much later (27). Mice were housed in groups of 4/cage in sterile polycarbonate mouse cages with filter tops (microisolator caging) with hardwood “beta” chips. Beta chips (Northeastern Products Corp) are sterilized wood particles used as contact bedding, processed from hardwood chips. They are heat-treated to reduce bacteria and enhance absorbency, and aspirated to remove dust.

Weights were measured each week. The nude and SENCAR mice were ascertained to be specific-pathogen free for the following agents: mouse hepatitis virus (MHV), Sendai virus (SeV), lymphocytic choriomeningitis (LCM), minute virus of mice (MVM), glycoprotein D (gD) of Herpes Simplex virus type 1 and type 2 virus, REO-3 virus, and ectromelia (ECTV). They were tested weekly for these virus by serology and histopathology. Autoclaved tap water was provided ad libitum in water bottles. Room temperature was maintained at 71°F ± 3°F with relative humidity between 30% and 70%. A 12-h dark/12-h light cycle was maintained. Mice were maintained in a 2-corridor barrier facility with a 1-way traffic pattern to ensure entry via a clean corridor and independent exit. Personnel assigned to the nude and SENCAR mice wore appropriate apparel including Tyvek (DuPont) coveralls, shoe covers, bouffant caps, latex gloves, and face masks. Mice were killed starting by asphyxiation with carbon dioxide vapors. Livers were removed and stored at –70°C until processed.

### Standard curves and recoveries

[15-^3^H]-Retinol in 100% ethanol was added to the biological samples to measure recovery of retinol and RP. These were found to be 85% for both. An aliquot of the final extracted sample was applied to HPLC, and the peak height of standard was compared with the theoretical peak height to calculate recovery.

### HPLC

A model 110 A pump (Beckman Instruments) connected to a Knauer variable wavelength detector (Sonntek) and a Radiomatic radioactivity flow detector high sensitivity (Radiomatic Instruments and Chemical Co.) were used. RP was eluted at 22′ and retinyl stearate at 26′, using a C-130 guard column (Upchurch Scientific) in series with a Waters C-18 (5 μm) “Resolve” column (3.9 mm i.d. × 30 cm). The detector was set at 325 nm and the mobile phase was acetonitrile:dichloromethane:methanol:1-octanol (90:15:10:0.1) plus 0.1% BHT (28) using a flow rate of 1.2 mL/min.

The analysis of retinol was performed on a Partisil 10 ODS-2 column (4.6 mm i.d. × 25 cm; Whatman, Inc.) fitted with a precolumn of Pellicular octadecylsilane (ODS) (Whatman, Inc.). A Beckman model 110A pump was connected to a Gilson 116 UV detector (Gilson Medical Electronic) and a Radiomatic radioactivity flow detector. The mobile phase was acetonitrile:1% ammonium acetate in water (65:35) (29).

### Extraction of retinoids from livers

Samples (0.5 g in 1 mL saline) were homogenized with a Polytron homogenizer (Brinkman Instruments). After the addition of 5 volumes of chloroform:methanol (2:1), samples were thoroughly mixed for 2 min and spun at 10,000 × *g* (3000 rpm) for 10 min at 25°C. The organic phase was removed, evaporated to dryness under a slow stream of nitrogen, and dissolved in 0.3 mL ethanol. All samples were filtered through a Microfilterfuge tube (Rainin Instrument Co.) with a 0.2-μm nylon filter and kept at –20°C until analyzed. Aliquots were taken for retinoid analysis.

### Immunohistochemistry

The procedure for the detection of K5-positive foci was described in references 30 and 31. Generally, groups of 4 mice were killed at specified times, and the entire reproductive system was removed and fixed quickly in 70% ethanol at 4°C. Paraffin-embedded sections (5 μm) were usually prepared (10 per mouse) to include the entire length of the epithelial lining from the vaginal *os* to the ovaries for immunohistochemical staining. Affinity-purified rabbit antiserum (32, 33) specific for keratin K5 was used for immunohistochemical staining. Rabbit serum was used as a control. The sections were exposed to biotinylated goat antirabbit secondary antibodies and the Vectastain ABS kit was used (Vector). Peroxidase staining was performed using Streptavidin-HRP Systems obtained from Kirkegaard and Perry Laboratories, Inc. The procedure used follows the original method (32). The Histomark staining system utilized 3,3′-diaminobenzidine (DAB) (Kodak). The working solution was identical to that described in reference 33.

### Statistical methods

Statistical comparisons of the weight between groups  were made using a 2-sample *t* test for unequal variances, using the square root of the animal weight. The RP levels between *nu/nu* and *nu/+* littermates were compared using the Wilcoxon rank-sum test (34). All *P* values are based on 2-sided tests.

Statistical comparisons of concentrations of retinol and RP between mouse strains were made while controlling for age by performing an ANCOVA. An ANCOVA model was constructed using the square root of nanograms all-*trans*-retinol (Rol)/gram as the response variable, mouse strain as the factor, and age (in weeks) as the covariate for mice ≥4 wk of age. A similar ANCOVA was performed using the square root of nanograms RP/gram as the response variable, mouse strain as the factor and age (in weeks) as the covariate for mice ≥4 wk of age. For each model, if strain was statistically significant in the ANCOVA model, Tukey's honest significant difference (HSD) test (35, 36) was performed to simultaneously test all pairwise comparisons of the 4 mouse strains to determine which of the strain pairs differed. All *P* values were assessed using a significance level of 0.05. Statistical analyses were performed, and graphs were generated using JMP^®^ version 16.0.0 (1989–2021; SAS Institute, Inc.).

## Results

### Response of immunodeficient female and male mice to a vitamin A–deficient diet

**FIGURE 1:**
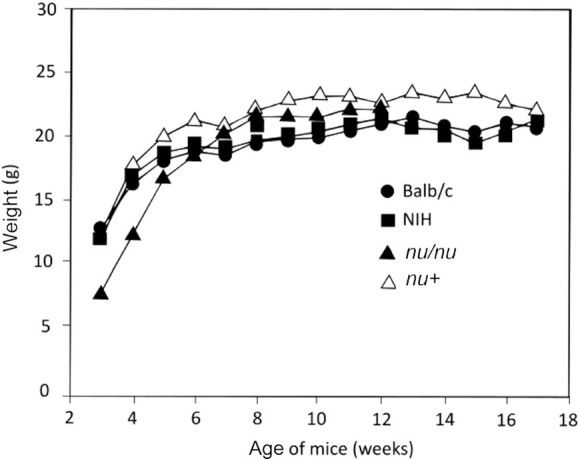
Body weights of the females of different mouse strains maintained on an RA− diet. Body weights were measured in groups of 4 mice housed in each cage between week 3 (weaning time) and week 17 of age. Measurements for the *nu/nu* mice were stopped at week 13 because of mortality.

**FIGURE 2:**
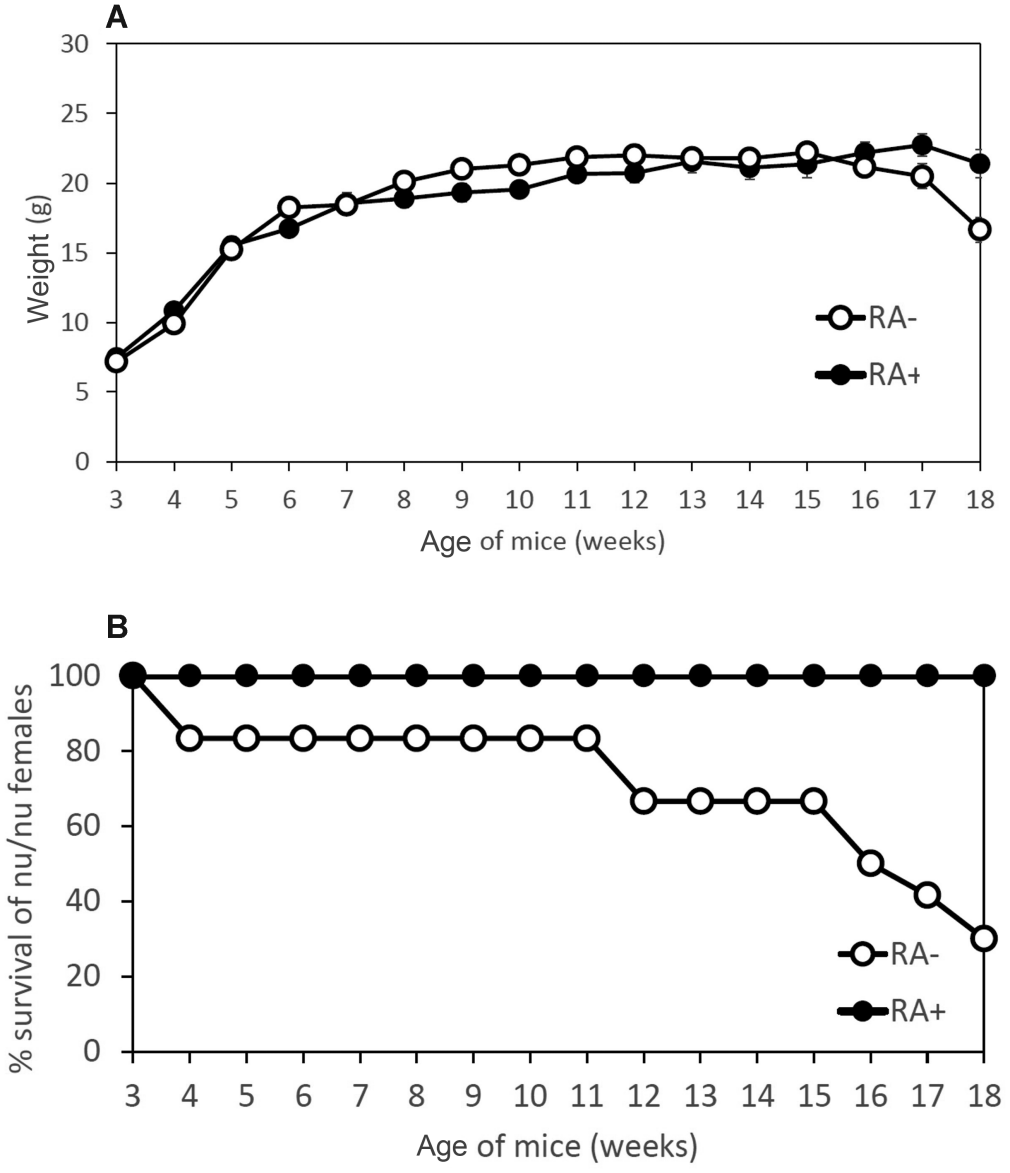
Body weights and survival of the females of *nu/nu* mice maintained on an RA+ or on an RA− diet. (A) Body weights of *nu/nu* female mice fed the RA+ (9 mice at week 3) and RA– (10 mice at week 3) diets. Comparisons were made using a 2-tailed *t* test for 2 samples with unequal variances. Error bars represent SEs. At week 18, the weights of the RA+ mice were significantly (*P < *0.01) greater than those of the RA− group. (B) Comparison of % survival of *nu/nu* female mice fed the RA+ (9 mice at week 3) and RA– (10 mice at week 3) diets.

**FIGURE 3:**
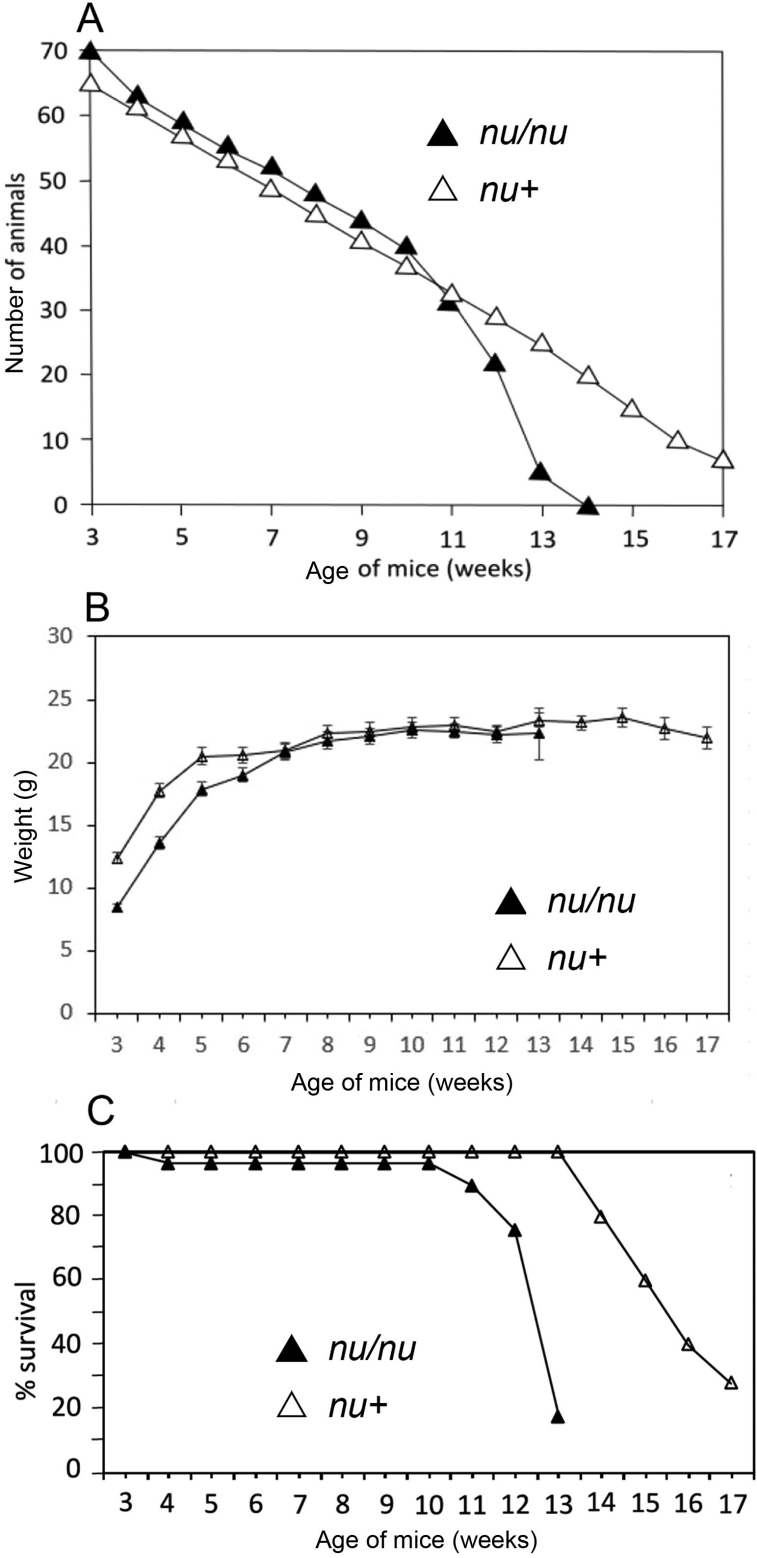
Body weights and survival of the females of *nu/nu and nu/+* mice maintained on an RA– diet. (A) Survival comparison between *nu/nu* and *nu/+* female littermates. The constant rate of decline (4 mice per week in both groups) represents utilization of these mice for retinoid measurement (Figures 6–9) and for immunohistochemistry (Figure 11 and Table 1). (B) Comparison of body weights between *nu/nu* (31 mice at week 3 of age) and *nu+* (25 mice at week 3 of age) female mice maintained on an RA– diet. Comparisons were made using a 2-tailed *t* test for 2 samples with unequal variances. Error bars represent SEs. The weights of the *nu/nu* mice could only be measured up to week 13 because of mortality. (C) Survival curves for *nu/nu* and *nu+* female mice maintained on a vitamin A–deficient diet.

**FIGURE 4:**
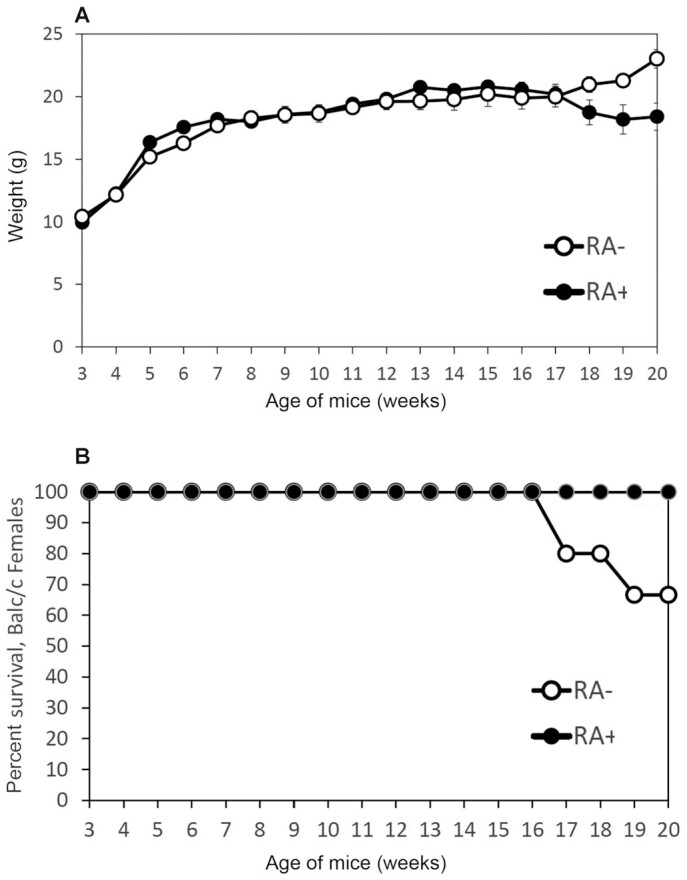
Body weights and survival of Balb/c female mice maintained on an RA+ or on an RA− diet. (A) Comparison of body weights in Balb/c female mice maintained on an RA+ (6 mice) or RA– diet (6 mice at week 3 of age). In weeks 5 and 6, the weight of RA− mice is significantly greater than RA+ (*P < *0.05). At week 20, RA+ mice were significantly heavier (*P < *0.01) than the RA– mice. (B) Percentage survival in Balb/c females mice kept on an RA+ or RA– diet.

**FIGURE 5:**
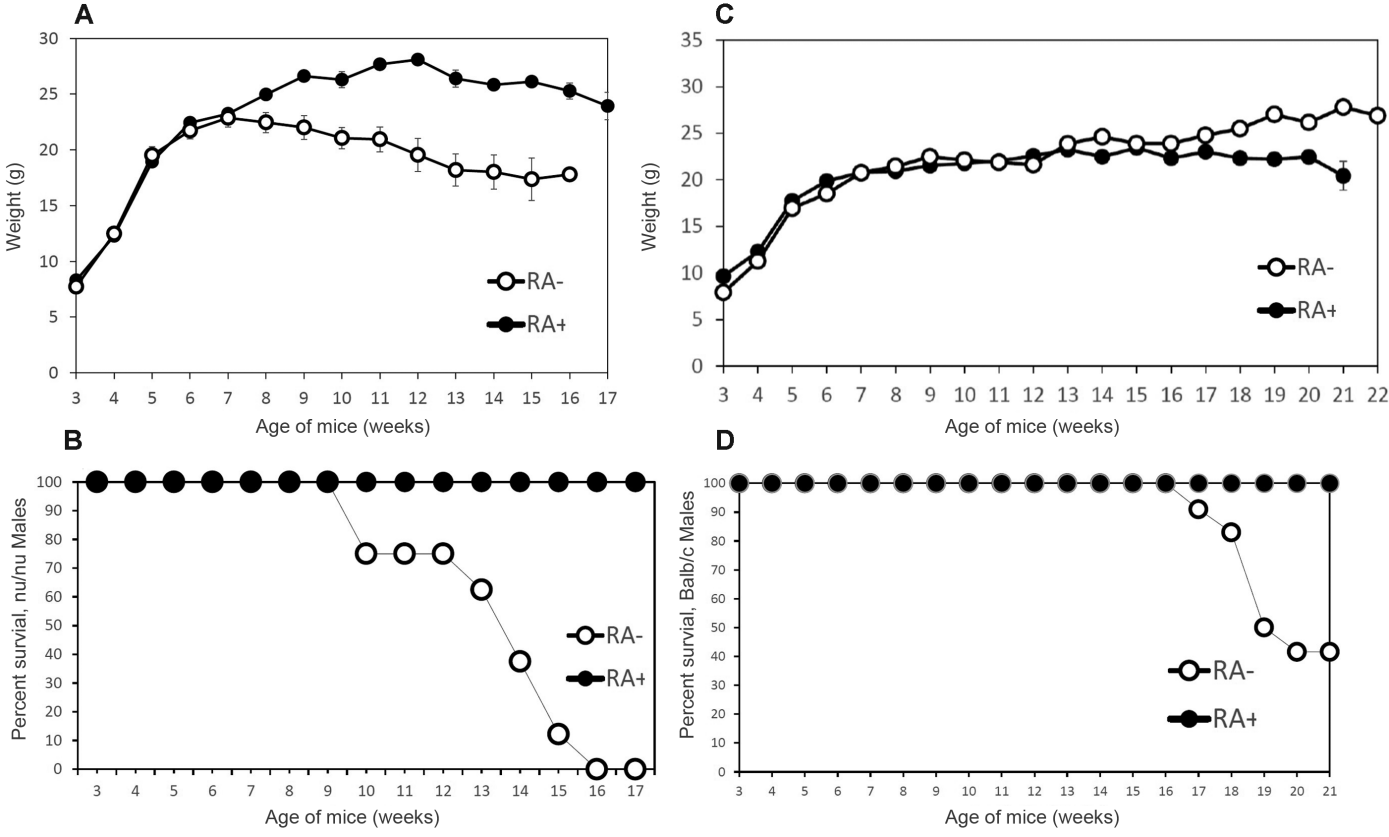
Body weights and survival of the males of different mouse strains maintained on an RA+ or on an RA– diet. (A) Body weights of *nu/nu* male mice maintained on an RA– diet (8 mice at week 3) or on an RA+ diet (9 mice). Comparisons were made using a 2-tailed *t* test for 2 samples with unequal variances. At week 8, the weight of the RA+ group is statistically significantly greater than that of the RA– group (*P < *0.05) and at weeks 9–14 at a highly significant level (*P < *0.01). Comparisons were made using a 2-tailed *t* test for 2 samples with unequal variances. Error bars represent SEs. (B) Comparison of percentage survival of *nu/nu* male mice (shown in Figure 2A) fed the RA+ and RA– diet. (C) Body weights of Balb/c male mice maintained either on an RA– (12 mice at week 3 of age) or on an RA+ (8 mice) diet. The weight of the RA+ group is statistically significantly greater (*P < *0.01) than that of the RA– group from week 17 to 21. Comparisons were made using a 2-tailed *t* test for 2 samples with unequal variances. Error bars represent SEs. (D) Survival (%) of Balb/c male mice (shown in Figure 5C for body weight) maintained either on an RA− or RA+ diet.

**FIGURE 6:**
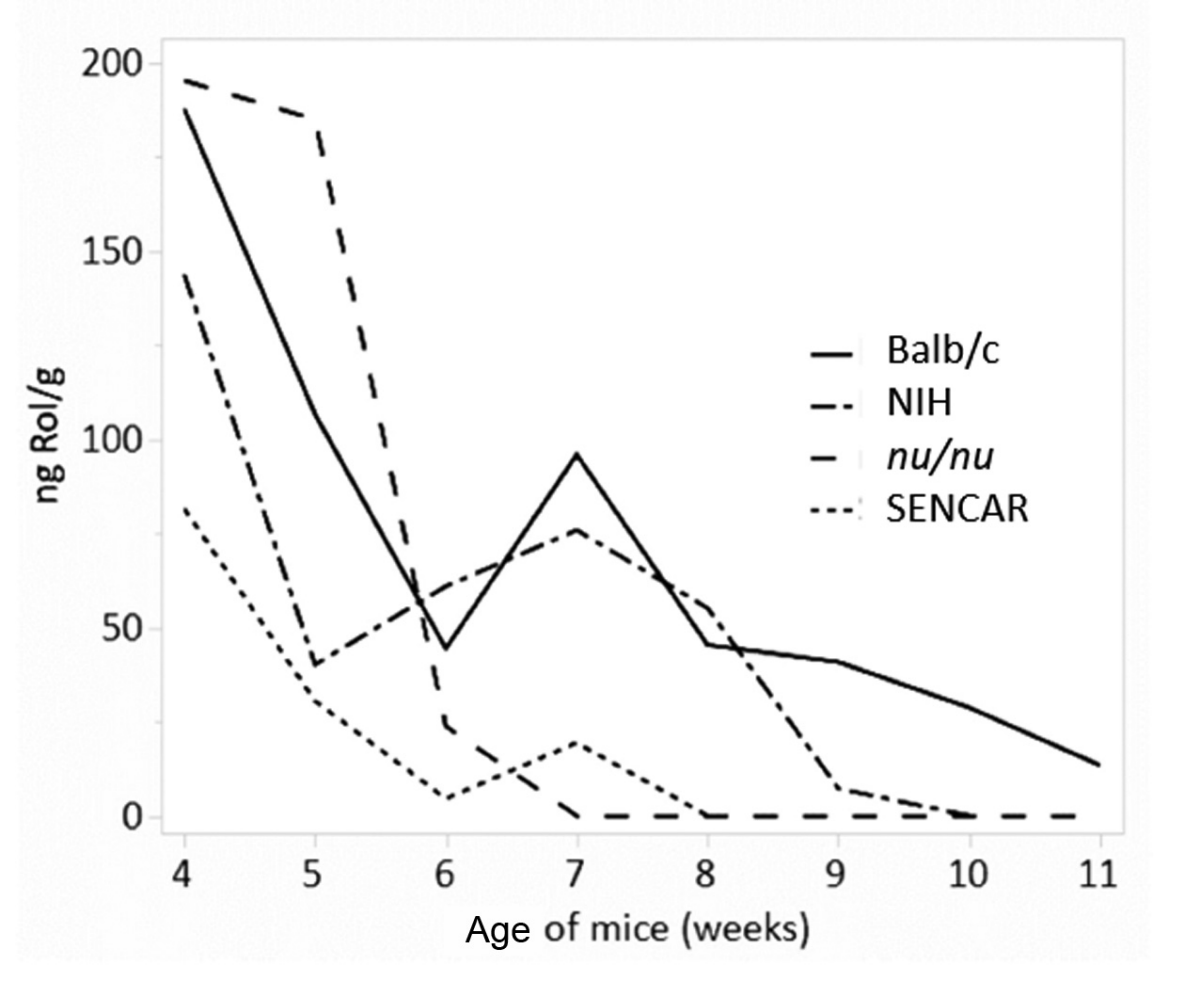
HPLC analysis of liver retinol. Line plot showing the mean retinol concentration over age (in weeks) of 4 mouse strains starting at week 4.

**FIGURE 7:**
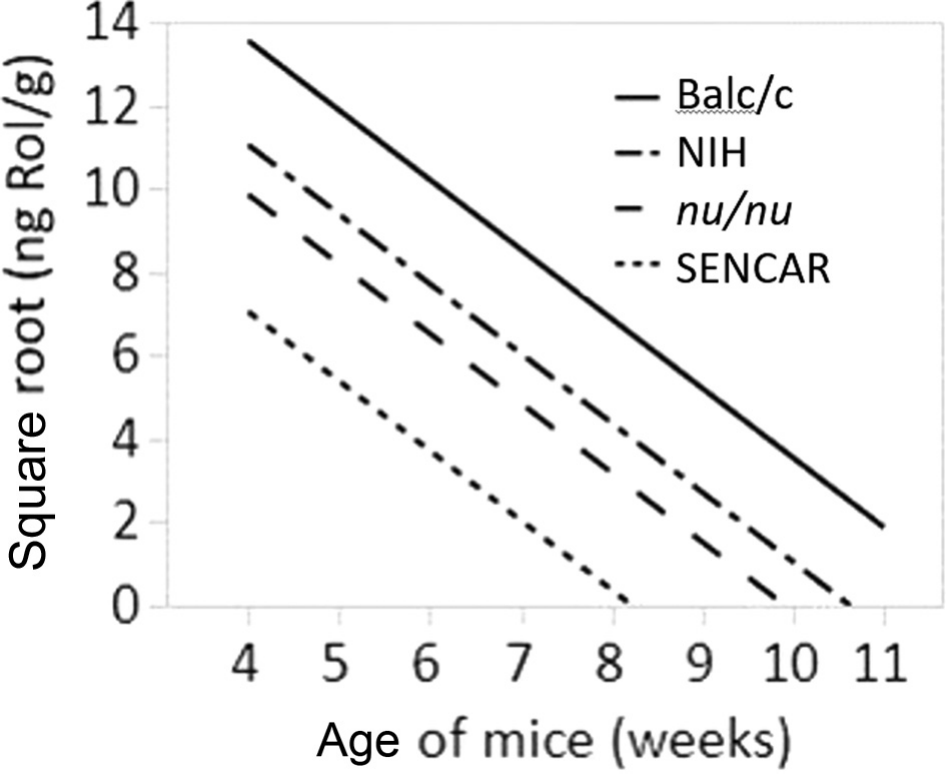
Regression plot for retinol. Regression plot from the ANCOVA of the square root of nanograms Rol/gram versus age (in weeks) for 4 different mouse strains ages 4–11 wk.

**FIGURE 8:**
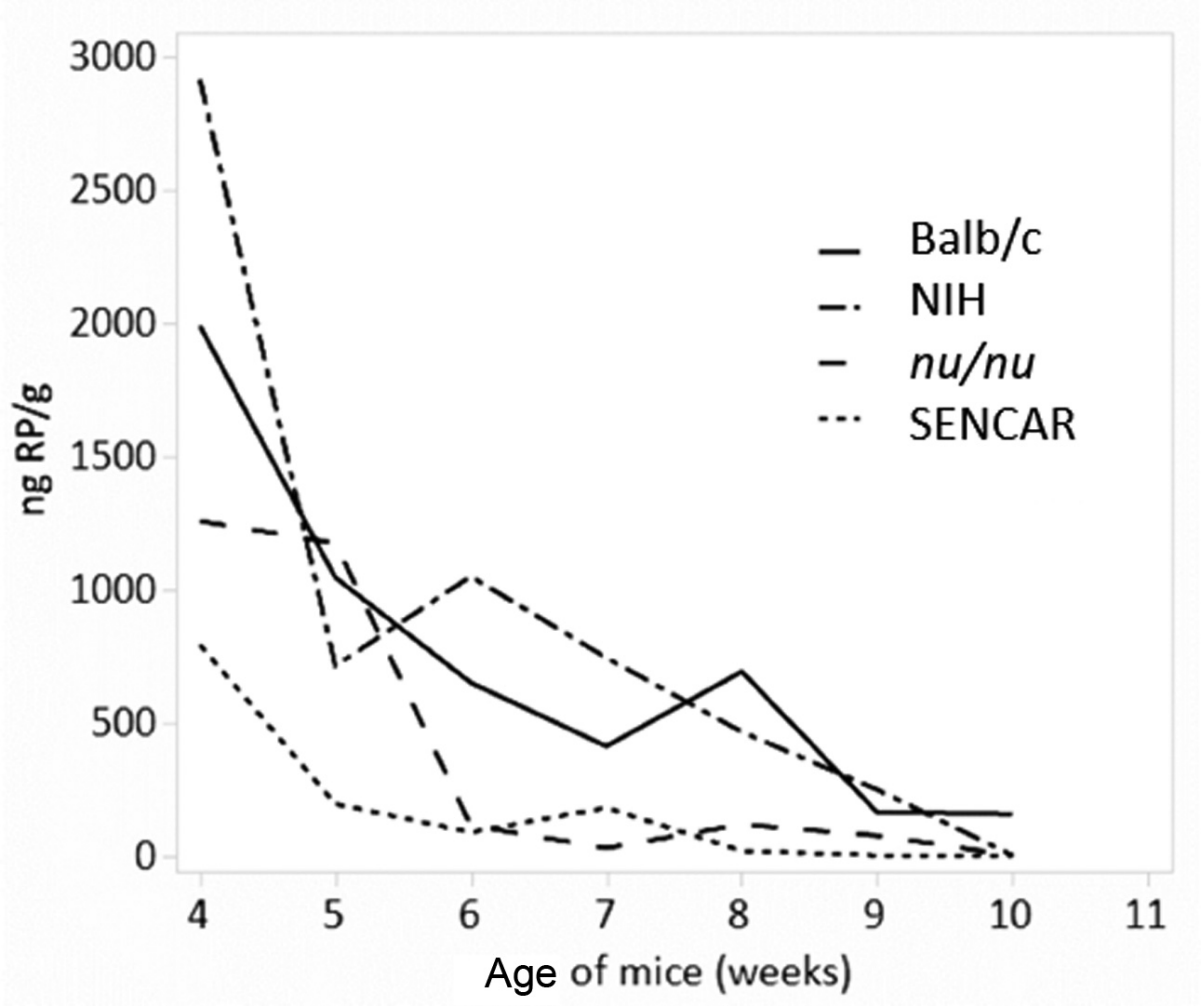
HPLC analysis of liver RP. Line plot showing the mean RP concentration over age (in weeks) of 4 mouse strains starting at week 4.

**FIGURE 9:**
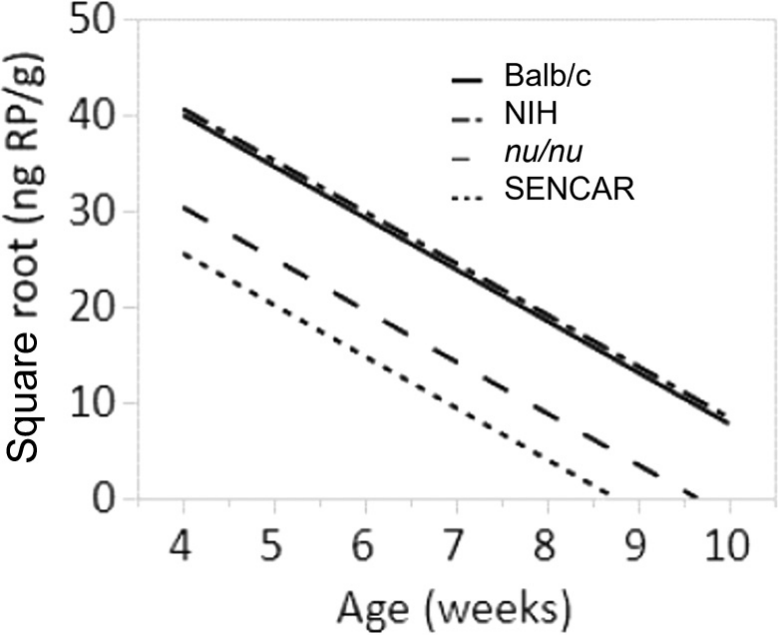
Regression plot for RP. Regression plot from the ANCOVA of the square root of nanograms RP/gram versus age (in weeks) for 4 different mouse strains ages 4–10 wk.

**FIGURE 10:**
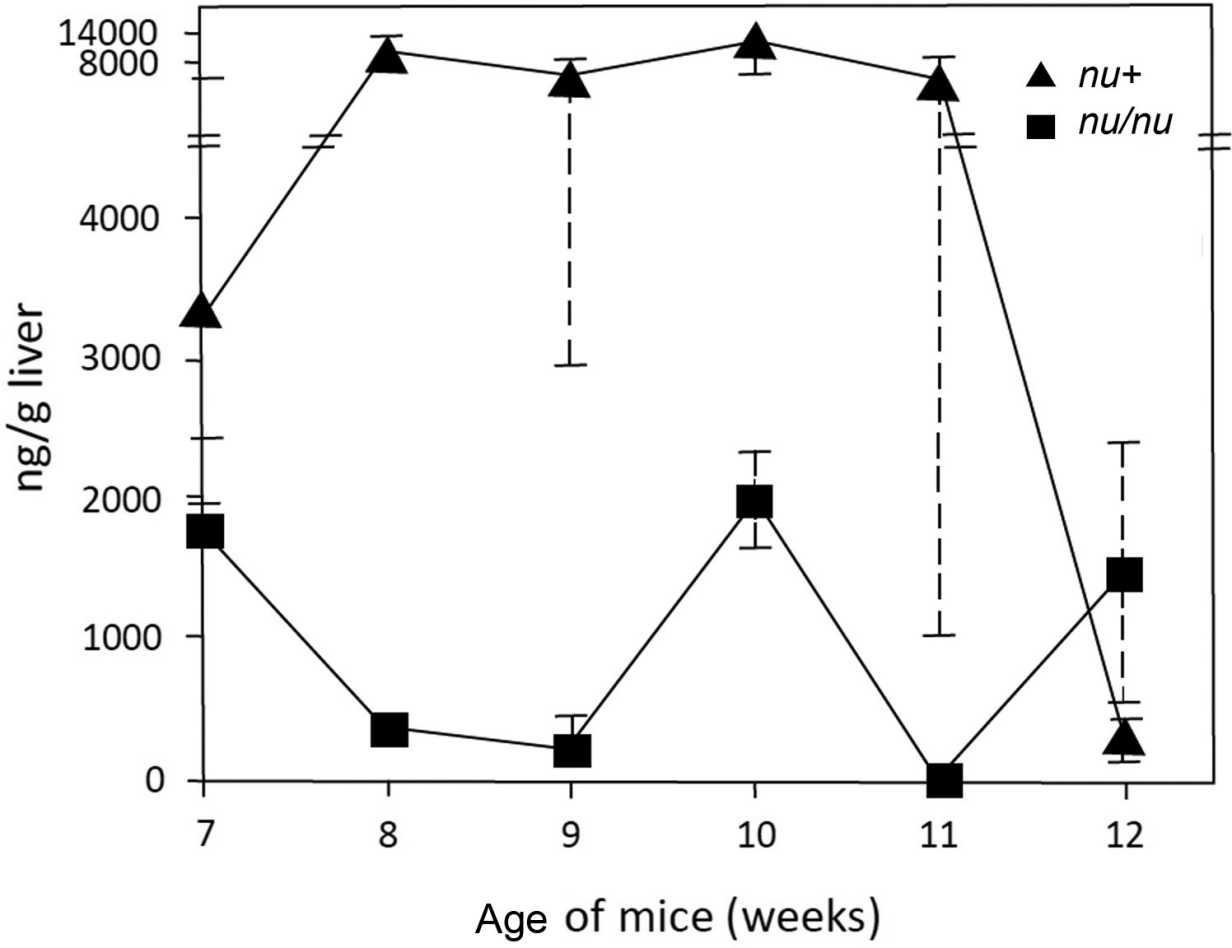
Comparison in RP concentrations between *nu/nu* and *nu/+* littermates. Mice in this experiment were weaned onto the vitamin A–deficient diet at week 4. Using the Wilcoxon rank-sum test, differences between homozygous and heterozygous mice were significant for week 8 (*P *< 0.001), week 9 (*P *= 0.013), and week 10 (*P *= 0.021). The *P* value for week 11 was 0.06.

**FIGURE 11:**
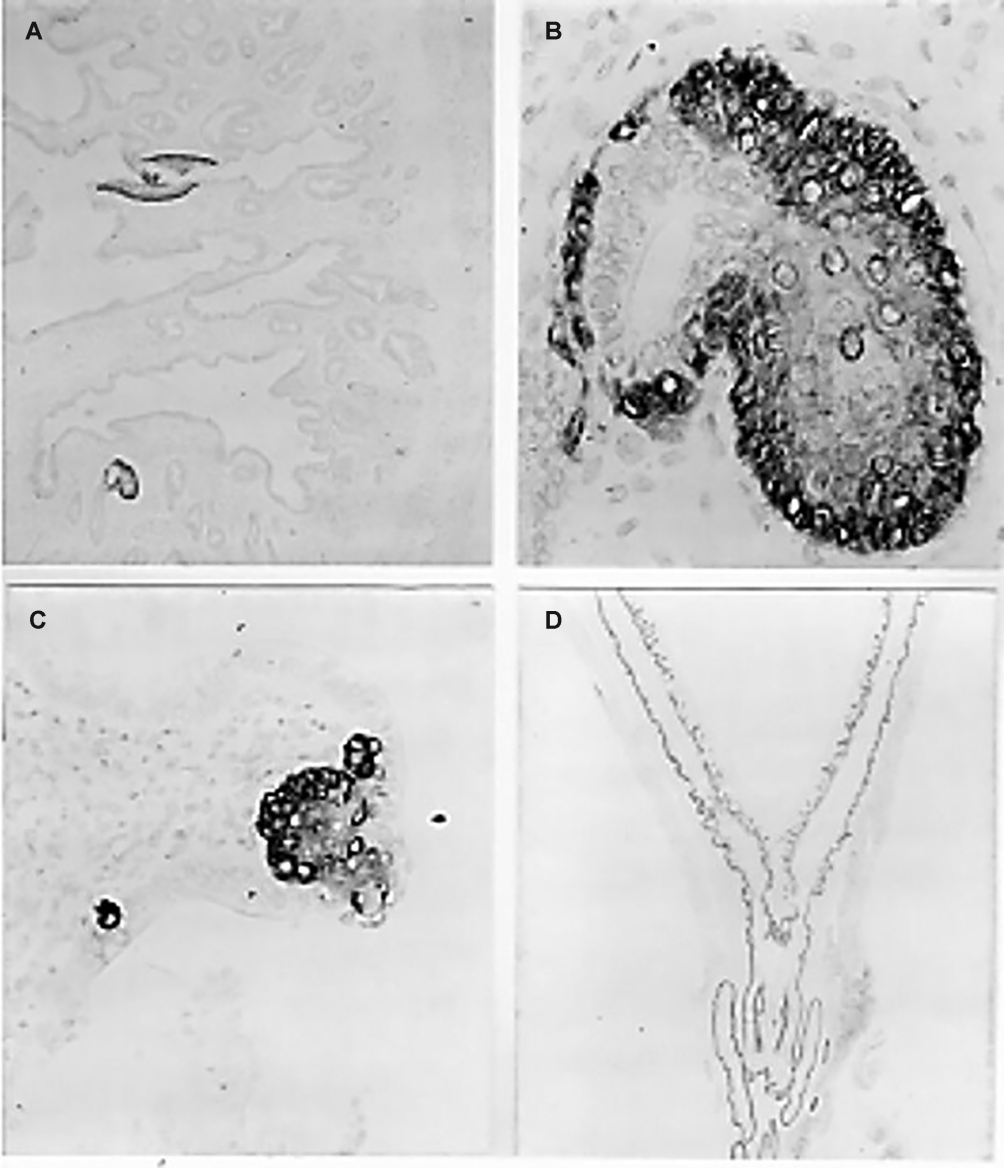
Monitoring of squamous metaplasia of the uterine epithelium by immunohistochemistry. Procedures were as described in the Methods section. (A, B) The heterozygous littermate *nu/+* mice showed squamous metaplastic foci staining for keratin 5 at week 13 (original magnification is 80× in panel A and 630× in panel B). (B) The stratified-squamous morphology of the metaplastic focus. (C) The nude (*nu/nu*) mice began to show squamous metaplastic foci at week 10 (original magnification, 50×). (D) Complete replacement of the simple columnar epithelium of the uterine glands with a stratified squamous epithelium, which stains with keratin-5 antibody, is clearly visible (original magnification is 6.5×) in all mouse strains surviving at 15 wk on the vitamin A–deficient diet. RA-fed mice failed to show any staining (not shown).

In **Figure 1**, the weight of the *nu/nu* female mice is shown up to week 14, because at week 15 all the *nu/nu* female mice had died due to vitamin A deficiency. The female *nu+*, Balb/c, and NIH mice exhibited longer survival and did not show significant loss of body weight. This was confirmed in additional experiments (e.g., **Figure 2**A and **3**B show body weight, and Figure 2B, 3A, and 3C show survival). In all these experiments the *nu/nu* females started to die at least 3 wk earlier than their *nu/+* counterparts. In female mice fed the diet containing 3 μg all-*trans*-retinoic acid (RA)/gram diet there was no difference in mortality between *nu/nu* and *nu/+* or other strains (Figures 2B, 3A, and **4**B). Comparisons of the weight between groups   are shown in Figures 2A, 3B, 4A, and **5**A and C. Although Figure 2A shows loss of body weight beginning at week 16 in the *nu/nu* female mice maintained on the vitamin A–deficient diet, most of the mice (75%) had already died without manifestation of loss of body weight. Although variability in survival was observed from experiment to experiment (compare, e.g., Figures 2A and 3B), the *nu/nu* mice always showed greater sensitivity to the deficiency regimen compared with the other mouse strains, and to the heterozygous counterparts, which started to die later (e.g., Figure 3C). Balb/c female mortality rates were similar to the *nu/+* female mice (Figure 4B). These mice lost weight significantly only at week 18 of vitamin A deficiency (Figure 4A). Statistical analysis of *nu/nu* and *nu+* female mice fed the deficient diet showed no significant differences in body weights (Figure 3B) in the last 7 wk of life, even though the *nu/nu* mice weighed less at the beginning of the experiment—indeed, a phenotypic characteristic of the *nu/nu* mice (17, 19). Female mice maintained on the same diet supplemented with 3 μg RA/g of diet showed similar body weights up to week 16 (Figure 2A) for *nu/nu* and for Balb/c (Figure 4A) mice. In the experiment in Figure 3A, also showing that death occurred earlier in the vitamin A–deficient *nu/nu* female mice than in the *nu+* vitamin A–deficient mice (Figure 3A), the constant rate of decline (average of 4 mice per week in both groups) represents the utilization of these mice for liver retinol and RP measurement (**Figures 6****–****9**). The RP levels in *nu/nu* decrease before those in the *nu/+* littermates as shown in **Figure 10**. The immunohistochemistry results can be seen in **Figure 11** and **Table 1**. The addition of RA at 3 μg/g diet prevented vitamin A deficiency and animal death in all the experiments.

**TABLE 1:** K5+ foci of squamous metaplasia appear earlier in the endocervix of *nu/nu* mice than *nu+* mice

	*nu*+											*nu/nu*					
*n*	4	4	4	4	4	4	4	4	4	4	4	4	4	4	4	4	4
Age, wk	5	6	7	8	9	10	11	12	13	14	15	5	6	7	8	9	10
Number of sections	40	40	40	40	40	40	40	40	40	40	40	40	40	40	40	40	40
K5+	0	0	0	0	0	0	0	0	40	30	40	0	0	0	10	0	30

In sharp contrast to the females, male *nu/nu* mice showed pronounced loss of body weight (Figure 5A) as well as increased earlier mortality (Figure 5B) when maintained on a vitamin A–deficient diet. Male *nu/nu* mortality started at week 10, and all male *nu/nu* mice had died by week 16 (Figure 5B). Similar to the females, Balb/c males were more resistant to vitamin A deficiency than *nu/nu* mice and showed a later loss of body weight due to deficiency than their *nu/nu* counterparts (i.e., starting at week 17) (Figure 5C), when mortality also began to be observed (Figure 5D).

In conclusion, in all of these experiments, whether in female or male mice, the immunodeficient *nu/nu* mice showed symptoms of vitamin A deficiency earlier than the *nu/+* or NIH or Balb/c mice.

We then extended these studies to a model of humoral immunodeficiency, the SENCAR mouse (26). These mice derive their name from the characteristic superior sensitivity to chemical and viral carcinogens (24, 37). When used in a similar dietary protocol, the female SENCAR mice showed early signs of vitamin A deficiency, including foci of K5-positive cells and 50% mortality within 12–13 wk of being fed the vitamin A–deficient diet (31) (i.e., between the very responsive *nu/nu* and the other mouse strains).

### HPLC analysis of liver retinol and RP

We compared the rate of liver retinoid disappearance in the 2 immunodeficient mouse strains, the *nu/nu* and the SENCAR mouse, with the rate of disappearance from the liver of the NIH and Balb/c mouse strains during the onset of vitamin A deficiency. A line plot showing the mean concentration over the age of the mice starting at week 4 (i.e., much before any observable change in survival) is shown in Figure 6.

Between weeks 4 and 6 the concentration of retinol declined rapidly for all mouse strains, as shown in Figure 6. Notably, by week 6, the nanograms Rol/gram in SENCAR and *nu/nu* mouse strains was much lower than that in the other 2 strains, and by 8 wk there was no measurable retinol in the immunodeficient strains (Figure 6).

To confirm that the rate of depletion of Rol differed between the immunodeficient and the other strains, an ANCOVA was performed using the square root of the nanograms Rol/gram as the response variable with mouse strain as the factor and age (in weeks) as the covariate for mice 4–11 wk of age. The ANCOVA results showed that there was a significant difference in nanograms Rol/gram between strains while adjusting for age (*P < *0.0001). The regression lines are shown in Figure 7.

To determine which of the strains differed, Tukey's HSD test was performed to simultaneously test all pairwise comparisons. The results indicate that all pairwise comparisons except for *nu/nu* vs. NIH (*P = *0.3383) were statistically different, with *P < *0.0001 for *nu/nu* vs. Balb/c, *P < *0.0001 for SENCAR vs. Balb/c, and *P < *0.0001 for SENCAR vs. NIH.

Since RP is by far the most abundant form of vitamin A in liver, we also measured this compound by HPLC analysis. A line plot showing the mean concentration of RP over the age of the mice starting at week 4 is shown in Figure 8.

To assess whether mean nanograms RP/gram concentrations declined at different rates for each strain, an ANCOVA was performed using the square root of the nanograms RP/gram concentration as the response variable with mouse strain as the factor and age (in weeks) as the covariate for mice 4–10 wk of age. The ANCOVA results showed that there was a significant difference in nanograms RP/gram between strains while adjusting for age (*P < *0.0001). The regression lines from the ANCOVA are shown in Figure 9.

To determine which of the strains differed, Tukey's HSD test was performed to simultaneously test all pairwise comparisons. The results indicate that Balb/c vs. NIH (*P = *0.9902) and *nu/nu* vs. SENCAR (*P = *0.1229) were not statistically significantly different, but all other pairwise comparisons were statistically significantly different (*P ≤ *0.0001).

Statistical evidence supports the conclusion that Rol and RP concentrations are lower in the female SENCAR mice relative to the NIH and Balb/c strains. Statistical evidence also supports the conclusion that RP concentrations are lower in the female *nu/nu* mice relative to the RP concentrations in the NIH and Balb/c strains, and that the Rol concentrations are lower in the female *nu/nu* mice relative to the Rol concentrations in the Balb/c strain for mice older than 4 wk.

Statistical evidence does not support the conclusion that Rol concentrations are lower in the *nu/nu* mice relative to the Rol concentrations in the NIH mice between the ages of 4 and 11 wk, although, as seen in Figure 6, the Rol concentrations in the *nu/nu* strain between 6 and 9 wk were much lower than the Rol concentrations in the NIH strain during the same time frame, suggesting that Rol was depleted more quickly in the *nu/nu* strain relative to the NIH strain.

In another experiment, RP concentrations were measured starting at week 7 of depletion in *nu/nu* compared with heterozygous *nu+* littermates. In this experiment (Figure 10), each point is derived from the average of 3 mouse livers assayed in duplicate. Statistical analysis shows the differences between *nu/nu* mice compared with *nu/+* mice to be significant for weeks 8 (*P = *0.0049), 9 (*P *= 0.013), and 10 (*P = *0.021) and not significant at week 11 (*P *= 0.06) and 12 (*P = *0.39). The week 12 results are consistent with *nu/+* mice getting closer to the level of deficiency of the *nu/nu* mice at this time point.

### Immunohistochemistry

Foci of K5-positive cells appeared in the heterozygous and in the Balb/c and NIH mice at week 13 of consuming the vitamin A–deficient diet (Figure 11A, B). In sharp contrast, these squamous metaplastic multilayered K5-positive lesions consistently appeared much earlier in the *nu/nu* mice (Figure 11C) and at week 12 in the SENCAR mice (31). Uniform replacement of the simple columnar epithelium of the uterine horns with a stratified-keratinizing epithelium typical of the skin and vagina was observed at week 15 on the deficient diet (Figure 11D) in Balb/c, NIH, and *nu+* mice.

Table 1 illustrates a study, limited to 10 wk for the *nu/nu* and 15 wk for the *nu*/+, in which the earlier appearance of K5-positive squamous metaplastic foci in *nu/nu* vs. *nu*/+ mice is documented.

## Discussion

Nutritional deficiencies have been implicated as etiological agents in the induction of immune deficiency (9, 10, 38). In this paper we have explored the opposite question by hypothesizing that, through immunodeficiency, which raises the risk of infection and inflammation, nutritional deficiency can be exacerbated because of a possible higher metabolism and consumption of vitamin A. Specifically, we asked the question of whether immune deficiency affects the rate of vitamin A depletion in the liver. The question has relevance given *1*) the ubiquitous environmental exposures to inflammation, *2*) the dependency of the immune response on specific immunomodulatory cells, *3*) the multiple essential roles for vitamin A in mounting immune response, *4*) the synergism of these factors that can increase severity and fatality of infections (38, 39), and *5*) the evidence of vitamin A deficiency as a public health problem (9, 10, 38). Although immunodeficiency in humans has been reported to coincide with a reduction in circulatory vitamin A (39), complicating factors, such as the use of different drugs including antibiotics, may influence plasma vitamin A concentrations (40).

The *nu/nu* mice became vitamin A deficient 3 wk earlier than their heterozygous littermates and the Balb/c and NIH mice. Vitamin A deficiency followed the same temporal order in all strains: liver retinol and RP depletion, followed by squamous metaplasia of the peripheral uterine epithelium, and eventual death. This observed earlier deficiency in the immunodeficient strains applied to male mice as well, as shown in Figure 5. A limitation of these studies is that liver vitamin A was measured instead of whole-body retinol and retinyl esters. Therefore, this must be taken into account in interpreting the results. The data are consistent with deficiency occurring earlier in the immunodeficient mice. Another limitation is that the *nu/nu* mice started at a lower body weight than the other strains. This would suggest a complexity of factors as contributors to the early onset of vitamin A deficiency in the immunodeficient *nu/nu* strain. Multiple pathologies have been studied in the *nu/nu* mouse model, including aspects of human tumor cell growth (19, 41), which can reveal potentially alterable mechanisms and pathways of clinical significance.

The SENCAR mouse has also been used extensively because of its susceptibility to either chemical (42) or virus-induced papilloma formation (37). SENCAR mice develop papillomas in the nasal mucosa when infected with the newly discovered MusPV1 tumor virus (37). Further, SENCAR mice fed a vitamin A–deficient diet developed splenomegaly earlier than both NIH and the Balb/c mice fed an equally deficient diet (43). Interestingly, the SENCAR mouse has also been used to study the chemopreventive activity of retinoids, both applied topically and given in the diet (42, 44, 45).

Interestingly (27), a delay of 1 d in switching the diet from laboratory feed pellets to a semisynthetic vitamin A–deficient diet may cause a delay of 4 wk in the onset of vitamin A deficiency. Thus, the variability in the observed onsets of deficiency, as observed in the experiments shown in Figures 1–4, may well be due to small differences in the switching schedule of the animals. However, although variable, the immunodeficient mice always showed an earlier (on average, 3 wk) response to the vitamin A–deficient diet.

The involvement of RA in several aspects of immune cell functionality and survival (46–49) may enhance the requirement for RA in the naturally athymic nude mice due to the necessity for other cells that are retinoid-dependent to take over the function of the deficient and missing thymus cells.

Our suggestion that vitamin A may work as a hormone (50, 51) found confirmation in the discovery that RA acts through nuclear receptors just as steroid hormones (52). The recent finding of hotspots for Vitamin-Steroid-Thyroid Hormone Response Elements half sites (e.g., AGGTCA) within switch regions of immunoglobulin heavy-chain loci suggests a direct influence of certain vitamins/hormones on B-cell class switch recombination (53). The most recent work showing that vitamin A deficiency causes immunoglobulin dysregulation, squamous cell metaplasia, infectious disease, and death supports the hypothesis stated in reference 54 that suggests a direct influence of certain vitamins/hormones on B-cell class switch recombination. The finding that immunodeficient SENCAR mice are also subject to accelerated vitamin A deficiency could also find its explanation in the concept that not only entire cells involved in immunoresponsiveness but also immunoglobulin molecules themselves, which are defective in SENCAR mice, would require more of this vitamin for their biosynthesis, thus possibly suggesting that both cellular and molecular mechanisms are dependent on this vitamin.

## Data Availability

Data described in the manuscript, code book, and analytic code will be made available upon request pending application and approval.

## Notes

Supported by NIH/Intramural NIH, Department of Health and Human Services.

Author disclosures: The authors report no conflicts of interest. Johns Hopkins University had no role in the study design and its implementation.

Abbreviations used: BHT, butylated hydroxytoluene; *Fox*, forkhead box; HSD, honest significant difference; NCI, National Cancer Institute; RA, all-*trans*-retinoic acid; Rol, all-*trans*-retinol; RP, retinyl palmitate.
